# *Limosilactobacillus reuteri* AJCR4: A Potential Probiotic in the Fight Against Oral *Candida* spp. Biofilms

**DOI:** 10.3390/ijms26020638

**Published:** 2025-01-14

**Authors:** António Rajão, João P. N. Silva, Diana L. Almeida-Nunes, Paulo Rompante, Célia Fortuna Rodrigues, José Carlos Andrade

**Affiliations:** 1 Associate Laboratory i4HB—Institute for Health and Bioeconomy, University Institute of Health Sciences—CESPU, 4585-116 Gandra, Portugal; antonio.rajao@iucs.cespu.pt (A.R.); nunesdiana@msn.com (D.L.A.-N.); jose.andrade@iucs.cespu.pt (J.C.A.); 2 UCIBIO—Applied Molecular Biosciences Unit, Translational Toxicology Research Laboratory, University Institute of Health Sciences (1H-TOXRUN, IUCS-CESPU), 4585-116 Gandra, Portugal; 3 UNIPRO—Oral Pathology and Rehabilitation Research Unit, CESPU, CRL, 4585-116 Gandra, Portugal; joaosilva_06@hotmail.com; 4 Differentiation and Cancer Group, Institute for Research and Innovation in Health (i3S) of the University of Porto, 4200-135 Porto, Portugal; 5 LEPABE—Laboratory for Process Engineering, Environment, Biotechnology and Energy, Faculty of Engineering, University of Porto, 4200-465 Porto, Portugal; 6 ALiCE—Associate Laboratory in Chemical Engineering, Faculty of Engineering, University of Porto, 4200-465 Porto, Portugal

**Keywords:** *Candida* spp., oral candidiasis, oral probiotics, antifungal activity, resistance, biofilms

## Abstract

Oral candidiasis is one of the most common infections in the immunocompromised. Biofilms of *Candida* species can make treatments difficult, leading to oral infection recurrence. This research aimed to isolate a *Lactobacillus* with anti-*Candida* effects from the oral cavity. An oral *Lactobacillus* was isolated in caries-free individuals. The best isolate was evaluated against *Candida* spp. planktonic and biofilm forms. The bacterial impacts on *Candida* biofilms’ adhesion to acrylic discs were analyzed through an in vitro test. *L. reuteri* AJCR4 had the best anti-*Candida* activity in the preliminary screening. Results were promising in both planktonic and biofilms, particularly with *C. albicans* SC5314 and *C. tropicalis* ATCC750, where no viable cells were detected when using the cell-free supernatant (undiluted). In *C. glabrata* ATCC2001 and *C. parapsilosis* ATCC22019 biofilms, reductions of 3 Log_10_ and more than 2 Log_10_, respectively, were noted when using a cell suspension of *L. reuteri* ACJR4 (10^8^ CFU/mL). On polymethyl methacrylate acrylic discs, the cell-free supernatant reduced *Candida* adhesion, resulting in no viable cell detection on the surface. In conclusion, *L. reuteri* AJCR4 demonstrated notable antifungal activity against *Candida* biofilms. This oral isolate and its postbiotic can be a potential alternative strategy to oral candidiasis, especially to treat recalcitrant infections.

## 1. Introduction

Oral candidiasis (OC) is one of the most common infections, and it is caused by commensal yeasts such as *Candida* spp. In particular circumstances, such as immunosuppression states or dysbiosis, *Candida* spp. can grow, leading to infections [1] that can also be deeply damaging and in some cases life-threatening [2,3]. OC can be responsible for innumerous symptoms in the oral cavity [4], like pain, dryness (xerostomia), and reduced palate sensitivity [1]. The diagnosis is based on a clinical evaluation of the lesions on the oral mucosa, which can be performed by biopsy or mucosal brushing for microbiological verification, to finally select the appropriated management [5]. Even though *Candida albicans* is still the major pathogen related to OC, non-*Candida albicans Candida* species (NCACs) are being increasingly reported, particularly *Candida glabrata, Candida parapsilosis*, and *Candida tropicalis* [6].

Biofilms are one of the main virulent factors responsible for the higher resistance in *Candida* spp. As complex microbial communities, these structures are highly organized and embedded in a rich polymeric matrix. Biofilms can delay the drugs’ access to *Candida* but also provide support and nutrients to the biofilm cells, which are also genotypically different from their planktonic counterparts. These features make it harder to eradicate infections and to prevent their escalation to more serious situations [7]. Furthermore, it is also known that the colonization by *Candida* biofilms on the acrylic resin of dental prostheses—which is associated with denture stomatitis—can act as reservoir of microorganisms. These situations happen more frequently in patients with poor oral hygiene, the elderly, and those who are highly medicated as well as in denture wearers [8,9]. Indeed, clinical resistance event rates have been increasing, and conventional antifungals are losing the battle to *Candida* infections [10,11].

Some of the conventional antifungal therapies can be divided into three main groups: azoles, polyenes, and echinocandins. Azoles are classified as five-membered compounds, which are divided into two groups. Imidazoles have two nitrogens in the azole ring, for example, clotrimazole, ketoconazole, and miconazole, with the latter used in OC. Triazoles have three nitrogens in the azole ring, for example, fluconazole, itraconazole, voriconazole, and posaconazole [12,13]. This antifungal class is used for the treatment and prevention of *Candida* infections by inhibiting the development of the infecting yeast and not allowing resistant species to evolve. *C. glabrata* is one of the species with a reduced susceptibility to azoles [1]. Regarding polyenes, amphotericin B and nystatin are the two drugs used to fight *Candida* infections. Their target is the plasma membrane where binding to ergosterol occurs, creating pores and causing leakage of cellular components and monovalent ions (K^+^, Na^+^, H^+^, and Cl^−^), leading to cell death [14]. Only amphotericin B is used in systemic treatments, and to minimize the toxicity of this treatment, topical versions are used in the oral cavity for a limited period of time [15]. Polyenes are also chosen when previous episodes of resistance have occurred [16,17]. Echinocandins are designed to disrupt and penetrate the fungal cell wall, the first line of defense of the fungus. They increase the permeability and consequently cause amino acid leakage, thereby exerting a fungicidal effect [18]. This class of antifungals, which was more recently developed in the 1990s, includes caspofungin, micafungin, and anidulafungin. Resistance events are also known and normally connected to point mutations [1]. In addition, these drugs have some side effects that cannot be overlooked, such as hepatic and renal toxicity [3,19]. As OC has become a major health problem, alternative strategies and therapies are crucial to overcome the problem, and probiotics and/or postbiotics can be an interesting solution to this issue [20].

According to the World Health Organization (WHO), probiotics are described as live microorganisms that confer beneficial effects to the organism when administrated in adequate amounts [21]. Oral probiotics have been widely used to help control the homeostasis needed in the oral cavity [22]. Some of the reported benefits include the regulation of the inhabitant flora, the inhibition of the adhesion of microorganisms by competing for adhesion sites and co-aggregating with them, the secretion of antimicrobial molecules, the modulation of the immune system, and maintaining a balanced microbiome [3,22]. *Lactobacilli* are a regular presence in the oral environment and gastrointestinal system and are a part of the human microbiome. Recent studies have showed that *Limosilactobacillus reuteri* and *Lacticaseibacillus rhamnosus* have a beneficial role in the human host and have already been used as probiotics in vulvovaginal candidiasis and OC, respectively [22,23]. In addition, postbiotics, produced by live bacteria, namely enzymes, acids, and peptides, among others, have been shown to help to maintain a healthy environment [24].

Thus, the aim of this study was to isolate and identify *Lactobacillus* with anti-*Candida* activity from the oral cavity of healthy subjects. The antifungal activity was evaluated and characterized against *C. albicans* and NCACs in both reference and oral clinical strains.

## 2. Results

### 2.1. Isolation and Selection of the Oral Isolates

**Figure 1:**
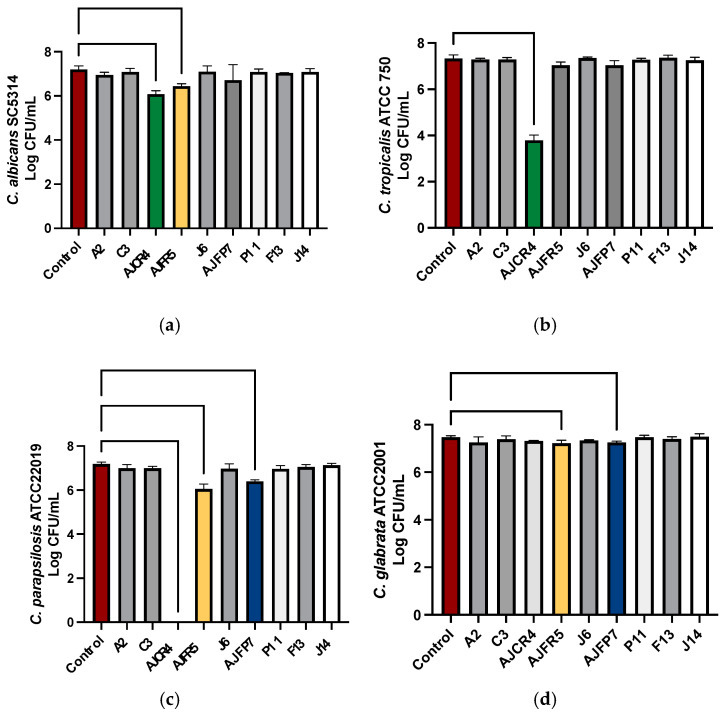
*C. albicans* SC5314 (**a**), *C. tropicalis* ATCC750 (**b**), *C. parapsilosis* ATCC 22019 (**c**), and *C. glabrata* ATCC2001 (**d**). CFUs after being co-cultured with *L. reuteri* and *L. rhamnosus* oral isolates. Mean, error bars indicate standard deviations.

From 38 volunteers preliminarily selected as potential donors, 24 were considered eligible to give saliva samples. After handling, from nine samples, we were able to isolate *Limosilactobacillus reuteri* and *Lacticaseibacillus rhamnosus* strains, all of them being Gram-positive and catalase-negative. For the initial screening, the co-incubation method (*Candida* spp. + isolate) was used. In this test, three isolates showed ability to reduce the growth of the four reference strains of *Candida* spp. (Figure 1): AJCR4 reduced the growth of *C. albicans* SC5314, *C. tropicalis* ATCC750, and *C. parapsilosis* ATCC22019, while AJFR5 inhibited *C. albicans* SC5314, *C. parapsilosis* ATCC22019, and *C. glabrata* ATCC2001. On the other hand, AJFP7 inhibited *C. parapsilosis* ATCC22019 and *C. glabrata* ATCC2001. These oral isolates were later identified as *L. reuteri* (AJCR4), *L. rhamnosus* (AJFR5), and *L. reuteri* (AJFP7).

Since *L. reuteri* AJCR4 showed the best results globally, it was selected to further characterize its antifungal activity.

### 2.2. Antifungal Activity of L. reuteri AJCR4 Against Candida spp. in Planktonic Form

**Figure 2:**
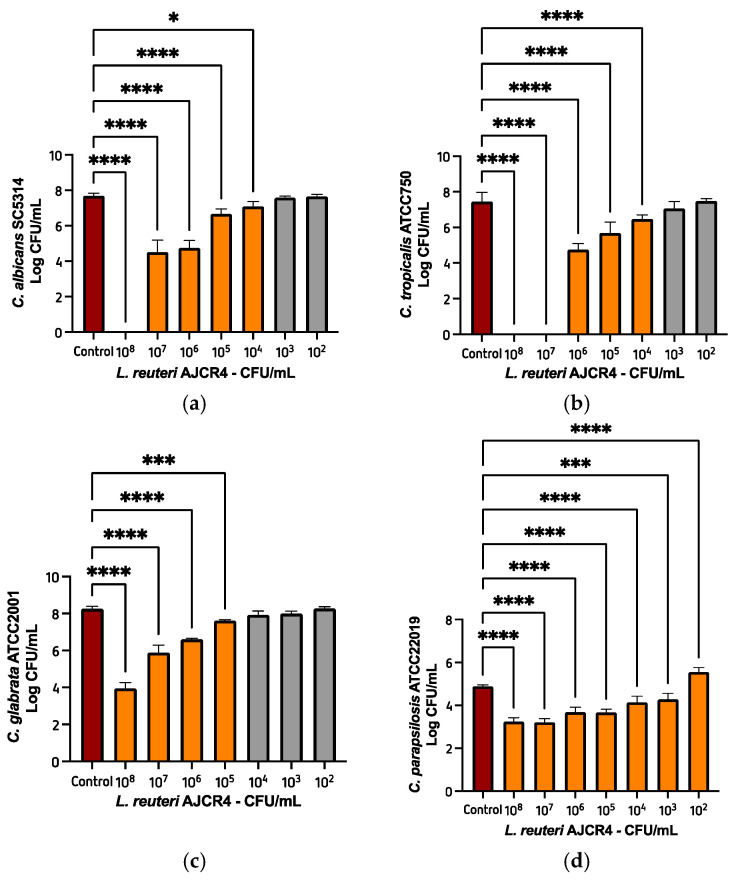
Cellular viability of planktonic cells of (**a**) *C. albicans* SC5314, (**b**) *C. tropicalis* ATCC750, (**c**) *C. glabrata* ATCC2001, and (**d**) *C. parapsilosis* ATCC 22019, treated with different concentrations of *L. reuteri* AJCR4. Bars represent the mean and standard deviation. (* *p* < 0.05; *** *p* < 0.001; **** *p* < 0.0001).

In order to characterize the antifungal activity of the cell suspensions of *L. reuteri* ACJR4, the strain was tested against planktonic cells of reference strains of *Candida* spp. using EUCAST methodology [25]. The oral isolate was tested using different concentrations ranging from 10^8^ CFU/mL to 10^2^ CFU/mL. At a concentration of 10^8^ CFU/mL of the *L. reuteri* AJCR4, no viable cells of *C. albicans* SC5314 were detected, while at 10^7^ and 10^6^ CFU/mL of bacterial suspension, a significative reduction (~3 Log_10_ and 2 Log_10_ CFUs, respectively) was observed (Figure 2a). Regarding *C. tropicalis* ATCC750, no viable cells were observed when a concentration of 10^8^ and 10^7^ CFU/mL of the bacteria was used, while at 10^6^ CFU/mL, a decrease of about 3 Log_10_ in the yeast was obtained (Figure 2b). In both strains, when the concentration of the bacteria was 10^3^ CFU/mL and 10^2^ CFU/mL, no reduction in growth was noticed.

*L. reuteri* AJCR4 seemed to be less effective against *C. glabrata* ATCC2001 and *C. parapsilosis* ATCC22019, though a significant reduction of 4 Log_10_ and ~2 Log_10_, respectively, was observed at a bacteria concentration of 10^8^ CFU/mL (Figure 2c,d).

The minimum fungicidal concentration (MFC) of *L. reuteri* AJCR4 obtained are presented in Table 1.

### 2.3. Antifungal Activity of Cells and Cell-Free Supernatant of L. reuteri AJCR4 Against Candida spp. Biofilms

**Figure 3:**
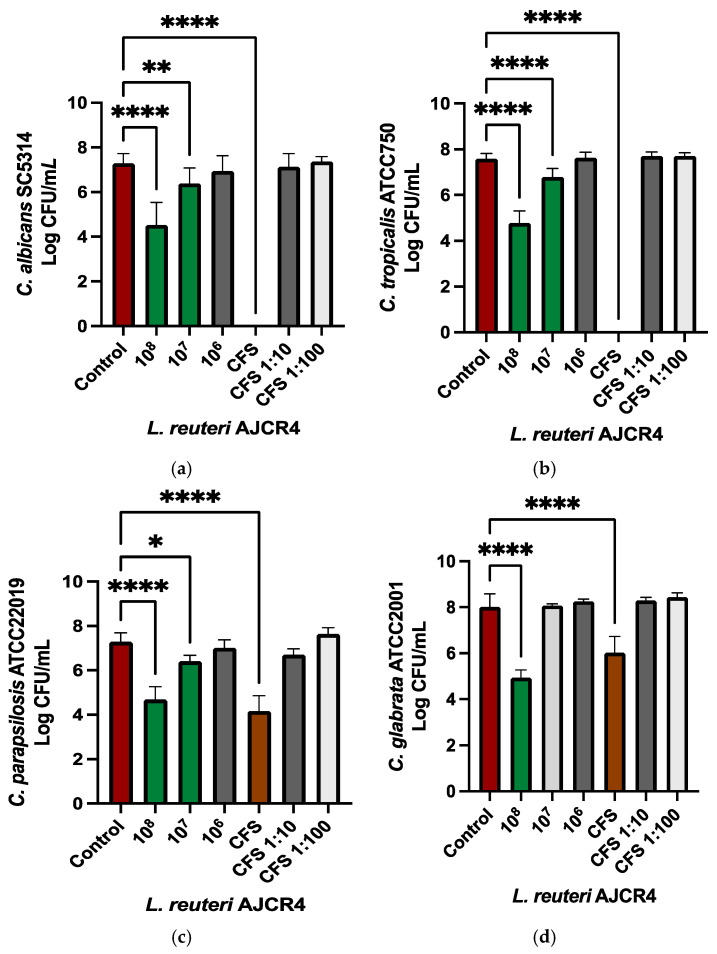
Biofilms cell reduction in (**a**) *C. albicans* SC5314, (**b**) *C. tropicalis* ATCC750, (**c**) *C. parapsilosis* ATCC 22019, and (**d**) *C. glabrata* ATCC2001 when treated with different concentrations of *L. reuteri* AJCR4 (10^8^, 10^7^, and 10^6^ CFU/mL) undiluted (CFS) and diluted 10 and 100 times (CFS 1:10 and CFS 1:100, respectively). Bars represent the mean and standard deviation (* *p* < 0.05; ** *p* < 0.01; **** *p* < 0.0001).

*Candida* biofilms are harder to fight, and thus, to assess the therapeutic potential, anti-biofilm activity must be evaluated. At a concentration of 10^8^ CFU/mL, *L. reuteri* AJCR4 cells showed a statistically significant reduction of ~ 2 Log_10_ in both *C. albicans* SC5314 and *C. tropicalis* ATCC750 (Figure 3a,b). Regarding *C. parapsilosis* ATCC22019 and *C. glabrata* ATCC2001, a reduction of 2 Log_10_ and 3 Log_10_, respectively, was observed with the same concentration of *L. reuteri* AJCR4 (Figure 3c,d). Undiluted cells-free supernatant (CFS) showed a large impact on *C. albicans* SC5314 and *C. tropicalis* ATCC750 biofilms since no viable cells were found on both strains (Figure 3a,b). For *C. parapsilosis* ATCC22019 and *C. glabrata* ATCC2001, CFS yielded a reduction of 3 Log_10_ and 2 Log_10_, respectively. However, when CFS was diluted 10 (CFS 1:10) or 100 times (CFS 1:100), no statistically significant reduction was observed in the tested *Candida* spp. (Figure 3c,d). The minimum biofilm eradication concentration (MBEC) of *L. reuteri* AJCR4 cells and CFS is presented in Table 2.

### 2.4. Antifungal Activity of Cells and Cell-Free Supernatant of L. reuteri AJCR4 Against Candida Oral Clinical Strains

**Figure 4:**
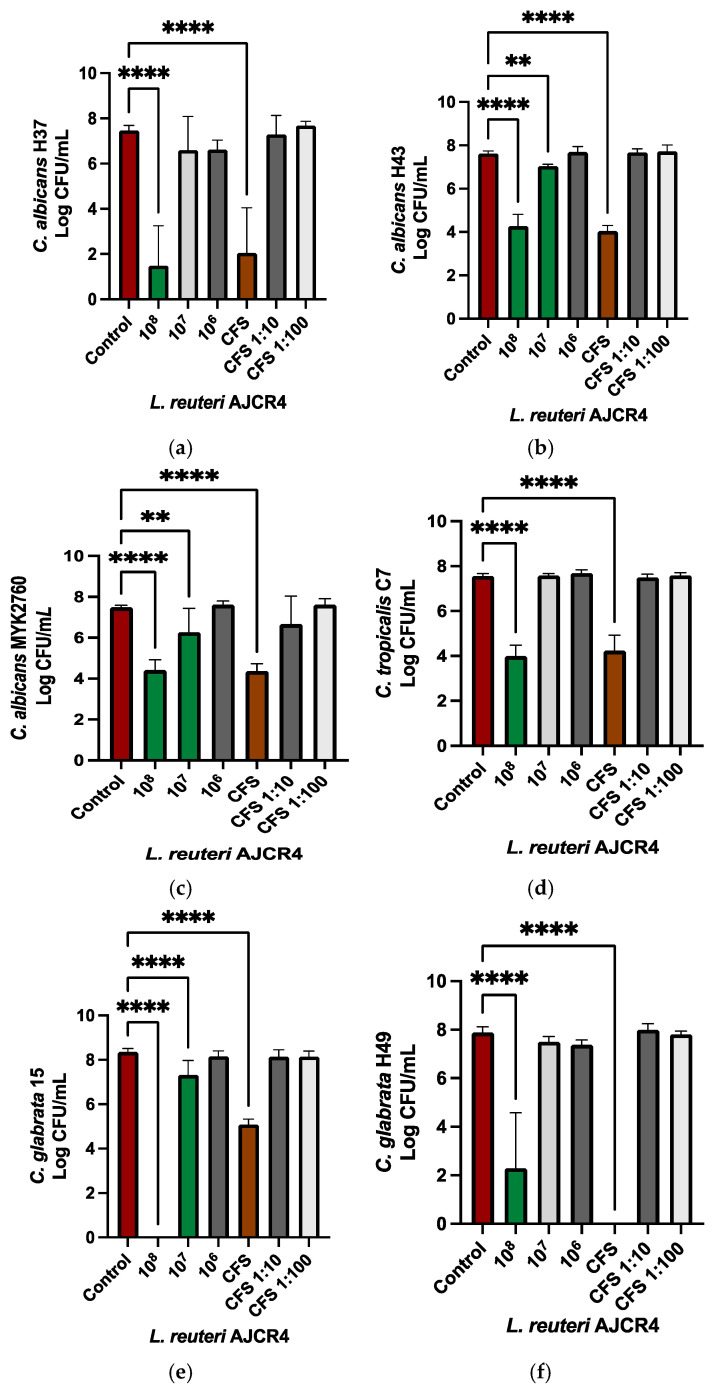
Biofilm reduction in (**a**) *C. albicans* H37, (**b**) *C. albicans* H43, (**c**) *C. albicans* MYK2760, (**d**) *C. tropicalis* C7, (**e**) *C. glabrata* H49, and (**f**) *C. glabrata* 15 when treated with different concentrations of *L. reuteri* AJCR4 (10^8^, 10^7^, and 10^6^ CFU/mL) and cells-free supernatant, undiluted CFS, and CFS diluted 10 and 100 times (CFS 1:10 and CFS 1:100, respectively). Bars represent the mean and standard deviation (** *p* < 0.01; **** *p* < 0.0001).

To further assess its therapeutic potential, the antifungal activity of *L. reuteri* AJCR4 was also evaluated against *Candida* oral clinical strains. The clinical strain with higher susceptibility to *L. reuteri* AJCR4 cells and CFS was *C. albicans* H37, which suffered a 6 Log_10_ and 5 Log_10_ reduction when challenged with bacteria cells (10^8^ CFU/mL) and CFS, respectively (Figure 4a). *C. albicans* H43 and MYK2760 and *C. tropicalis* C7 also presented a significative reduction of ~3 Log_10_ for both the bacteria cells and CFS (Figure 4b–d). Regarding *C. glabrata* H49, a reduction of 5 Log_10_ was observed with *L. reuteri* AJCR4 cells, and no viable cells were detected when CFS was used (Figure 4e). For *C. glabrata* 15, with 10^8^ CFU/mL of *L. reuteri* AJCR4, no viable cells of the yeast were found, but when the CFS was applied, there was a reduction of 3 Log_10_ (Figure 4f).

When diluted, neither cells nor CFS demonstrated a statistically significant reduction in yeast growth, except for *C. albicans* H43 and MYK2760 and *C. glabrata* 15 (~1 Log for 10^7^ CFU/mL) (Figure 4b,c,f).

### 2.5. Evaluation of the Impact of L. reuteri AJCR4 Cell-Free Supernatant on Adhesion and Inhibition of Candida Biofilms Production on Polymethyl Methacrylate Acrylic Resin Discs

**Figure 5:**
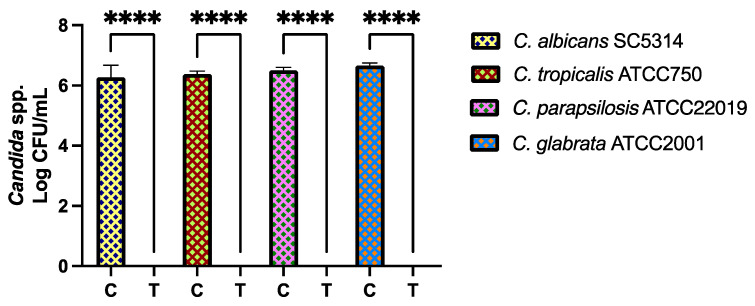
Viable biofilm cells of *C. albicans* SC5314, *C. tropicalis* ATCC750, *C. parapsilosis* ATCC 22019, and *C. glabrata* ATCC2001. The biofilms were formed on the acrylic discs treated with *L. reuteri* AJCR4 supernatant and their corresponding controls with MRS broth medium. **C** (control group) and **T** (treatment) of each *Candida* spp. with *L. reuteri* AJCR4 CFS. Bars represent the mean and standard deviation (**** *p* < 0.0001).

The in vitro effect of the CFS in the adhesion and production of *Candida* biofilms in acrylic resin discs was also assessed. On the four reference strains of *Candida* used, no viable cells were detected on the specimens submitted to treatment with CFS of *L. reuteri* AJCR4 (Figure 5). It is, though, important to note that the control showed normal *Candida* growth when not in contact with the CFS (i.e., the control positive had normal *Candida* biofilm formation).

### 2.6. L. reuteri AJCR4 Auto-Aggregation and Co-Aggregation

**Figure 6:**
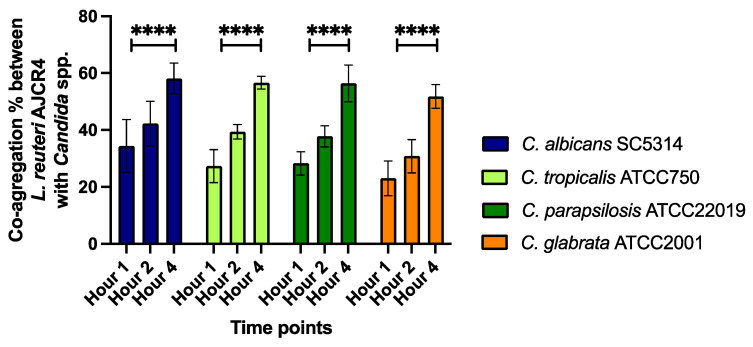
Percentage of co-aggregation among each *Candida* reference strain with *L. reuteri* AJCR4, at three time points (1, 2, and 4 h). Bars represent the mean and standard deviation (**** *p* < 0.0001).

*L. reuteri* AJCR4 auto-aggregation and co-aggregation, throughout four hours, were quantified. The ability of *L. reuteri* AJCR4 to auto-aggregate increased with time. The highest percentage of auto-aggregation was 44.35%, observed at 4 h. The results also showed that the co-aggregation increased gradually through time for all strains in the study. Indeed, after 4 h, the percentages of aggregation were 58.1% with *C. albicans* SC5314, 56.65% with *C. tropicalis* ATCC750, 56.35% with *C. parapsilosis* ATCC22019, and 51.78% with *C. glabrata* ATCC2001 when compared with 34.38%, 27.28%, 28.28%, and 23%, respectively, after 1 h (Figure 6).

### 2.7. Impact of Various Treatments on the Antibiofilm Activity of Cells-Free Supernatants

**Figure 7:**
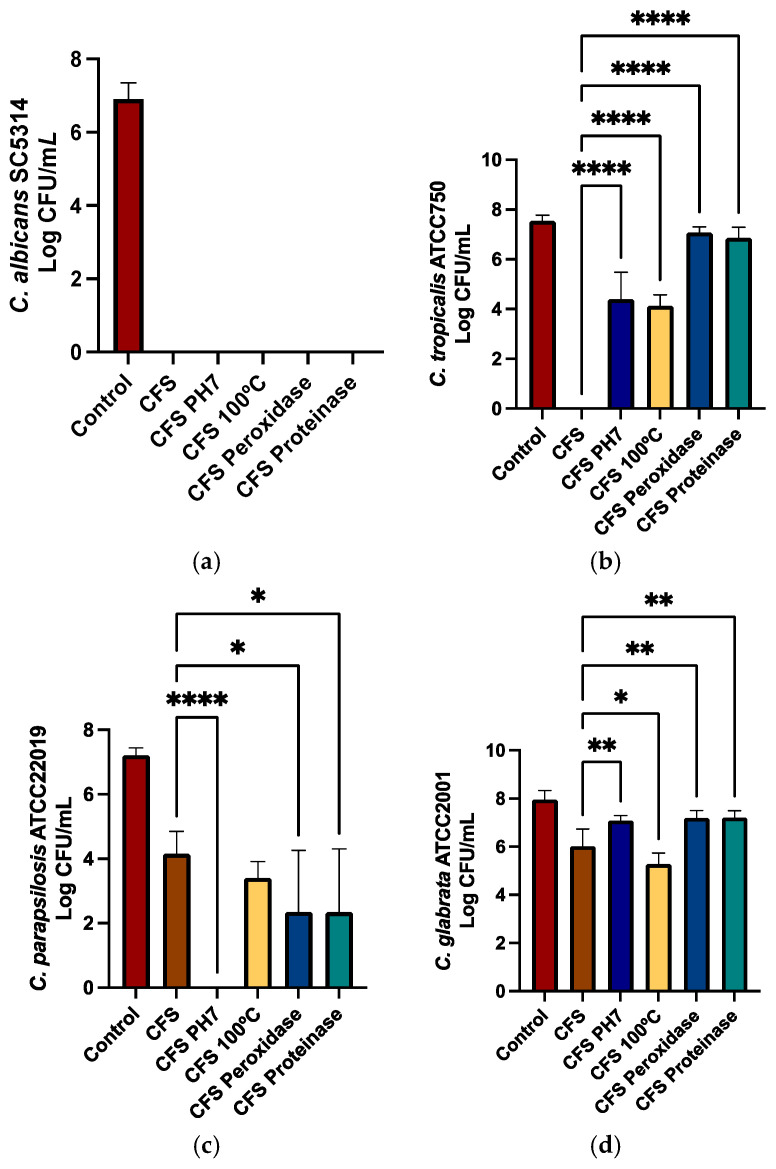
Antifungal activity of the cell-free supernatant both untreated (CFS) and with neutralization of pH (CFS pH7), heat treatment to 100 °C (CFS 100 °C), and enzyme treatments (CFS peroxidase; CFS proteinase) in (**a**) *C. albicans* SC5314, (**b**) *C. tropicalis* ATCC750, (**c**) *C. parapsilosis* ATCC 22019, and (**d**) *C. glabrata* ATCC2001. Bars represent the mean and standard deviation (* *p* < 0.05; ** *p* < 0.01; **** *p* < 0.0001).

To verify if the CFS maintained its activity, tests of pH neutralization, heat, peroxidase, and proteinase K were performed. After these assays, the CFS maintained its activity on *C. albicans* SC5314 biofilms, with no viable cells detected (Figure 7a). Regarding *C. tropicalis* ATCC750, when the CFS was pH neutralized or heated, a decrease in the antifungal activity was observed (Figure 7b). Nevertheless, a reduction in viability was observed compared to the control (*Candida* cells without CFS). After the peroxidase and proteinase K treatments, there was a loss of antifungal activity (Figure 7b). In *C. glabrata* ATCC2001 biofilms, there was a decrease in the CFS antifungal activity except for after the heat treatment. In this case, there was a decrease of ~1 Log_10_ in the CFU count when compared with non-treated CFS (Figure 7d). With the exception of heat, all treatments improved CFS activity against *C. parapsilosis* ATCC22019 (Figure 7c), particularly in the pH neutralization, where no viable cells were detected.

## 3. Discussion

The severity and recurrence of *Candida* spp. infections in the oral cavity are rising. The fight against these infections is now harder, and the therapeutic options are limited [10]. The occurrence of oral thrush has been associated with different species of yeasts (e.g., *C. albicans, C. parapsilosis*, *C. tropicalis*, and *C. glabrata*), which makes the treatment tougher, as different species require different treatments [26]. OC typically presents as creamy white lesions on the tongue, inner cheeks, gums, or throat, often accompanied by redness and soreness [27]. The oral cavity is known to be inhabited by a high number of microorganisms. Among them, *Lactobacillus* spp. can have beneficial activities against oral pathogens like *Candida* spp. [3,28,29,30]. *L. reuteri* is a lactic acid bacteria found in the human oral and gastrointestinal microbiome [31,32]. Indeed, *Lactobacillus* is one of the first probiotics identified, which can be isolated from humans; it primarily inhabits the human gastrointestinal tract as a symbiotic resident [33,34]. In this study, the method described in Rossoni et al. [22], was used to obtain several isolates; from the nine obtained, two were identified as *L. reuteri*, including *L. reuteri* AJCR4. In another report, using another methodology, four strains of *L. reuteri* were also isolated from the saliva sample of a caries-free volunteer [35].

The level of organization of *Candida* cells is extremely connected to the rate of success of therapies [36], so the characterization of an antifungal agent normally starts from lower organization levels, such as planktonic forms, to more complex ones. When tested against *Candida* spp. planktonic cells, *L. reuteri* AJCR4 eliminated all cells (no viable cells detected) of *C. albicans* SC5314 and *C. tropicalis* ATCC750 at a concentration of 10^8^ CFU/mL. With the same concentration, the isolate caused a reduction of 4 Log_10_ and ~2 Log_10_ in *C. glabrata* ATCC2001 and *C. parapsilosis* ATCC22019, respectively. Other authors also reported the anti-*candida* activity of *L. reuteri* strains using different methodologies and usually against a reduced number of *Candida* spp. Such is the example of Kohler et al. [37], who reported an inhibitory activity of *L. reuteri* RC-14 against *C. albicans* SC5314 (disk diffusion method). In another study, *L. reuteri* also inhibited *C. glabrata* ATCC2001C [38]. Furthermore, strains of *L. reuteri* (CRL1324 and CRL 1327) proved to restrain the growth of isolates of *C. albicans* and *C. tropicalis* [39]. Bakhshi et al. [23] tested *L. reuteri* at different concentrations against five *Candida* spp. isolated from oral cavity of HIV/AIDS patients, including *C. albicans*, *C. tropicalis*, *C. parapsilosis*, *C. glabrata*, and *C. krusei*. At a concentration of 10^8^ CFU/mL of lactic acid bacteria, no colonies were detected in all *Candida* strains tested. It should be noted that Bakhshi et al. used the agar overlay interference technique, while we used a co-incubation technique adapted from the EUCAST methodology.

Testing new approaches for new treatments against *Candida* biofilms is clinically relevant, as the biofilms are linked to substantial therapeutic concerns because of their higher virulence, resistance to conventional therapeutics, and invasion of host tissues [7]. To reach the stage of an organized biofilm, different levels of maturation are required, and the higher the maturation degree of the biofilm, the more repellent it is against stress, antifungal treatments, and host immunity [36]. In general, *C. albicans* SC5314 has a biofilm with a higher number of entangled hyphae, which can optimize the stability of the biofilm (when compared with NCAC) [2,7]. Moreover, when we fight biofilms, the number of viable biofilm cells is critical. It is known that biofilm cells are genetically and phenotypically very different (e.g., more tolerant or resistant to drugs) when compared to lower organized states like planktonic cells. Furthermore, the decrease in the biomass of a biofilm formation is usually but not mandatorily associated with less resistance to antifungals [40,41]. Indeed, there is a huge possibility of having viable biofilm cells that are recalcitrant to treatments and have a high ability to reform biofilm. This is why it is important to count the number of biofilm cells in order to determine their susceptibility to an antifungal agent.

Cell suspension of *L. reuteri* AJCR4 was related to a reduction in the *Candida* biofilm cells. *C. glabrata* ATCC2001 was the most susceptible, with a reduction of 3 Log_10_. In the remaining reference strains, a reduction of ~2 Log_10_ was detected. These results are important because most of the studies carried out to date have been focused on the CFS of *L. reuteri* rather than cell suspension of the bacteria. When the CFS of *L. reuteri* AJCR4 was used on *C. albicans* SC5314 biofilms, no viable cells were detected. These results align with the work of Boahen et al. [42], where the CFS supernatant of two strains of *L. reuteri* yielded a remarkable reduction in the biofilm of *C. albicans* ATCC14053 (±90%). The same report also showed a worthy reduction (>80% CFUs, ~2 Log_10_) in *C. glabrata* ATCC2001 biofilms with the same bacterial strains’ CFS. This may be explained by the fact that *C. glabrata* ATCC2001 has a biofilm constituted by a matrix richer in proteins and carbohydrates compared to *C. albicans*, which makes a more solid biofilm [42,43]. In the case of *C. tropicalis* ATCC750, the biofilm commonly has a very rich extracellular matrix that surrounds groups of cells, but it also can form hyphae [43]. Like in *C. albicans* SC5314, the AJCR4 CFS showed a stronger inhibition effect (no viable cells detected) when compared with *L. reuteri* AJCR4 cells. It is quite possible that this may be due to the antifungal compound (or compounds) consumption by *L. reuteri* attributed to its own metabolism activities [44]. Another possible explanation could be that the supernatant constituents could penetrate more easily through the hyphae into the biofilms in absence of bacterial cells. *C. parapsilosis* ATCC22019 biofilm comprises pseudo hyphae and a dense mantle with a high quantity of carbohydrates, the latter also being present in the biofilm of *C. glabrata* ATCC2001 [7,43,45]. These characteristics may explain why the inhibitory effect of AJCR4 and CFS cells is more similar (approximately 2 to 3 Log_10_), which means that diffusion is not a determining factor in inhibition for these two strains. Our data seem to point out that the effect of bacteria in *Candida* depends on the strain and the type of biofilm.

*Candida* spp. can have different behaviors depending on the strain, the type of isolate, gene mutations, from whom and from where it was collected, and its exposure to external factors [2,5,11,26,46]. Knowing the potential therapeutic effects against a variety of yeasts is required to ensure alternatives to resistance outbreaks. Therefore, the effects of AJCR4 cells and CFS were investigated against several clinical oral isolates of *Candida* spp. Alves et al. [46] demonstrated that *C. albicans* H37 and H43 (isolates from HIV patients, with antifungal resistance profiles) were resistant to azoles and showed moderate formation of biofilms. Nonetheless, our results showed that the isolate caused a reduction of around 5 Log_10_ for *C. albicans* H37 and around 3 Log_10_ for *C. albicans* H43, even when cell suspension and CFS were used, which is an interesting result. Other isolates, namely *C. albicans* MYK2760 and *C. glabrata* 15, were previously described as heavy biofilm producers with a matrix poor in polysaccharides and proteins quantities, the latter strain with superior values and more polysaccharides in proportion to proteins. Moreover, these two strains were shown to be resistant to antifungals such as fluconazole, voriconazole, anidulafungin, and amphotericin B [2]. *C. glabrata* 15 showed to be very sensible to cell suspension of *L. reuteri* AJCR4 (no viable cells), which is a significant result due to the potential difficulty in treating this strain. *C. albicans* MYK2760 had an inhibition of around 3 Log_10_ with both cell suspension and CFS of *L. reuteri* AJCR4 [2,11]. Alves et al. [46], showed that biofilm production by *C. glabrata* H49 was not prominent, which may explain why AJCR4 cell suspension and CFS was so effective on the biofilm disruption; the supernatant of *L. reuteri* AJCR4 led to no detectable viable cells, and the cell suspension caused a 5 Log_10_ reduction. This is also a relevant result since H49 was shown to have resistance to anidulafungin [46], an echinocandin and first-line therapy for systemic candidiasis [47].

The environment of the oral cavity of denture users is altered, enabling the installation of *Candida* sp. biofilms [48,49]. To further assess the therapeutic potential of AJCR4, the effect on the adherence of *Candida* species onto the denture base material (PMMA) was investigated. PMMA is commonly the selected material to manufacture removable dental protheses and repair full and partial dentures, but its surface can be a place where microorganisms accumulate as biofilms (e.g., *Candida)*. Dental protheses are a reservoir for *Candida* because the yeast can adhere to the acrylic resin, and without hygiene habits, biofilm formation is easier. Ishikawa et al. [50] showed that some lactic acid bacteria can diminish the oral cavity colonization of *C. albicans*, *C. tropicalis*, *C. parapsilosis*, and *C. glabrata* in denture wearers. Using commercial formulations of probiotics, Shankar et al. [51] demonstrated that adhesion of *C. albicans* in PMMA discs was reduced, thus showing that probiotics can be used as complementary therapy against *Candida* denture stomatitis. In addition, Catanoze et al. [8] also showed that a commercial formulation of *L. reuteri* NCIMB 30242 resulted in no decrease in the concentration of *C. albicans* ATCC 26790 on the acrylic resin specimens. These outputs are in agreement with the results of this study, where it was shown that the CFS of *L. reuteri* AJCR4 prevented biofilm adhesion of *C. albicans* SC5314, *C. tropicalis* ATCC750, *C. parapsilosis* ATCC22019, and *C. glabrata* ATCC2001, as no viable cells were found on the surface of the acrylic discs.

One of the initial steps of the colonization of *Candida* infection is its adhesion to a surface. Nonetheless, when this process cannot occur, *Candida* can be effortlessly dispatched, which results in the barring of yeast colonization [44]. Probiotic bacteria, such as *L. reuteri,* have the ability to compete for cellular adhesion of pathogenic microorganisms, which can be seen as a defense mechanism in developing infections, leading to yeast removal [23,52]. Bakhshi et al. [23] demonstrated that *L. reuteri* co-aggregated with different *Candida* spp., and after four hours, *C. tropicalis* had the higher percentage of co-aggregation, followed by *C. parapsilosis*. In this study, it was also demonstrated that *L. reuteri* AJCR4 had the ability to co-aggregate with *Candida* spp. The behavior between the four *Candida* reference strains and the bacteria was similar, with percentages of co-aggregation ranging from 23% to 58% after four hours of incubation time. Similarly, Jørgensen et al. [53] showed that two strains of *L. reuteri* co-aggregate with *Candida* strains, with percentages values between 30% and 40% [23,53]. Co-aggregation capacity allows the bacteria to connect with pathogenic microorganisms, hindering the establishment of these pathogens [54]. Some oral bacteria and other lactic acid bacteria have also been shown to co-aggregate with some pathogens, and this, combined with the production of some compounds, can be a significant protection mechanism against infections [55]. Auto-aggregation allows bacteria to accumulate enough mass to create biofilms and adhere to surfaces [56]. Studies have shown that the auto-aggregation ability of lactic acid bacteria made a beneficial contribution to the gastrointestinal microbiome [57] and that *L. reuteri* can adhere to mucosal surfaces and epithelial cells [54]. In this study, *L. reuteri* AJCR4 showed to have auto-aggregation that reached 44% after 4 h of incubation. Other researchers have also shown similar auto-aggregation percentages. Ali et al. [54] demonstrated that *L. reuteri* PSC102 had the ability to auto-aggregate to around 30% after 4 h of incubation, with increasing percentages as the incubation time progressed. Dell’Anno et al. [58] found that an isolate of *L. reuteri* also showed approximately 30% auto-aggregation after 4 h.

CFS can be composed of a variety of elements produced by bacteria growth, and the action of these [59] can be greatly different, depending on the bacteria responsible for its production, the pathogen with which the probiotics are interacting, and the environment. In order to gain some insight into the nature of the metabolites present in AJCR4 CFS, the effects of several treatments, such as pH normalization, heat, and enzymes, on CFS antifungal activity were investigated. The activity against pathogens by CFS is altered by variations in the pH [60,61]. As a lactic acid bacteria, *L. reuteri* can produce organic acids such as acetic, lactic, or phenylacetic acid, one of the main antifungal metabolites [57]. Boahen et al. [42] revealed that when *L. reuteri* 29A CFS was neutralized to pH 7, the anti-*C. albicans* and anti-*C. glabrata* strains were lost, resulting in the loss of the inhibition of the biofilms, which was clearly related to the acid neutralization. Chantanawilas et al. [62] also demonstrated that neutralizing the CFS of *L. reuteri* ATCC PTA 6475 had no effect against *C. albicans* ATCC 90028 and *C. tropicalis* ATCC 13803, meaning that these two species are sensitive to an acidic environment. Regarding *C. glabrata* ATCC 66032, the inhibition was maintained. This was linked to the fact that neutral and alkaline pH can fragilize cell wall integrity and, consequently, cell growth [62]. When *L. reuteri* AJCR4 CFS was neutralized, the activity against *C. albicans* SC5314 remained unchanged, while a reduction in inhibitory activity was observed in *C. tropicalis* ATCC750 and *C. glabrata* ATCC2001. This seems to indicate that the inhibition of *C. glabrata* ATCC2001 is probably due to organic acid production.

In general, lactic acid bacteria cultures that reach lower pH values often display higher inhibition rates. Nonetheless, this does not happen in all situations. In fact, sometimes, a low pH value reflects a direct inhibition of the fungi, which indicates that acid production is not the only reason for the antifungal effect of *L. reuteri* [39]. Unexpectedly, pH neutralization of AJCR4 CFS resulted in a higher inhibition of *C*. *parapsilosis* ATCC22019. The mechanism related to this result is presently not fully elucidated; therefore, further investigation is necessary.

It is acknowledged that the composition of *L. reuteri* supernatants is diversified, and protein compounds are one of the constituents. To verify if a compound of this nature has influence on the antifungal activity, the CFS of *L. reuteri* AJCR4 was heated to ensure if any substance was affected. When heated, the *L. reuteri* AJCR4 CFS yielded a loss of antifungal inhibition for *C. tropicalis* ATCC750, which may indicate that some substance(s) with protein biochemical characteristics and/or (other compound with) heat lability might be involved. For *C. glabrata* ATCC2001, an increase in inhibition was shown, which seems to indicate that what is responsible for its decrease is not heat sensitivity. No loss of antifungal activity was observed against *C. albicans* SC5314. This resistance to high temperatures suggests that proteins maybe not be totally involved in the antifungal activity but rather secondary metabolites or small peptides. Boahen et al. [42], observed that, after heating the CFS, the antifungal effects of *L. reuteri* 29A were not changed when tested on *C. albicans* and *C. glabrata* isolates compared with untreated CFS.

Enzyme treatments like proteinase k and peroxidase are used to help identify the nature of the compounds in the CFS and distinguish between protein-based antimicrobial compounds and hydrogen peroxide. Boahen et al. [42] demonstrated that, after treating the *L. reuteri* 29A CFS with catalase and proteinase K, the antifungal effects against the tested *C. albicans* strains remained, which corroborates our results with *C. albicans* SC5314. These results indicate that the inhibitory effect on *C. albicans* SC5314 is not due to H_2_O_2_ or any protein. On the other hand, when tested against *C. glabrata* strains, the results differed from ours. In our study, treating *L. reuteri* AJCR4 CFS with proteinase k or peroxidase led to the loss of antifungal effects. It should, however, be noted that in order to perform the enzyme treatments, a pH-neutralization step is required, which may explain the loss of antifungal activity, as the reduction in the yeast was of the same magnitude as the pH treatment (Figure 7d). In the case of *C. tropicalis* ATCC750, enzyme treatment resulted in some loss of antifungal activity when compared with the untreated CFS (Figure 7b). This may indicate that a combination of acidic and proteinaceous compounds maybe responsible for the action against this strain.

A comprehensive conclusion about the antifungal activity of *L. reuteri* AJCR4 is that a synergy between cellular effects and several compounds released by this bacteria can be attributed to the activity against *Candida* spp. AJCR4 CFS showed that the activity against *Candida* biofilms cannot be attributed to only one compound. As Grande et al. [63] and Vitale et al. [64] showed, *L. reuteri* DSM 17938 CFS has a complex composition [65]. Like other probiotics, the mechanisms behind this role can be attributed to competition for binding locations, immunomodulation, or production of postbiotic substances. *L. reuteri* is known to produce short-chain fatty acids and hydrogen peroxide [32,33,66,67,68,69] but also some low-molecular-weight substances, such as carbon dioxide or ethanol [70]. Some *L. reuteri* strains can form membrane vesicles that distribute substances responsible for probiotic activity [44]. As a matter of fact, the antimicrobial activity of probiotic bacteria CFS against pathogens from oral biofilms is recognized and associated with production of bioactive compounds, for example, bacteriocins, biosurfactants, and antimicrobial peptides, which can cause a reduction in the growth of the pathogens [64,71]. Among others, there is a metabolite that is produced by *L. reuteri* that shows antifungal activity and is known as 3-hydroxypropinaldehyde (3-HPA) [72], also identified as reuterin. Reuterin is a known postbiotic substance with reported antimicrobial activity [73]. *L. reuteri* can live in a low-pH environment [32,67] and has ability to produce lactic and acetic acids, with antimicrobial effects [67,68]. This can be seen at the initial pH value (3.93) of *L. reuteri* AJCR4 CFS. An acidic medium showed an influence on *L. reuteri* against microorganisms, especially when lactic acid was involved [61]. In a similar work, Schmidt et al. [74] demonstrated that phenyllactic acid, an antimicrobial compound with a wide spectrum, can be synthetized by *L. reuteri* R29, and a connection between this acid and the antifungal activity of this bacteria was observed.

The effects of the different bioactive compounds present on the CFS of lactic acid bacteria, namely *L. reuteri*, can diversify depending on the strain of the bacteria, the substrate, and the type of pathogens. In this study, different species of *Candida* were susceptible to different substances, which was also dependent on the interaction of the substances present on the CFS. These results show that AJCR4 cells and/or CFS may have clinical relevancy, even though deeper studies must be conducted before considering its potential use, such as increasing the number of isolates, complete characterization of the CFS, and safety evaluation.

Despite the limitations of the present exploratory study, the results demonstrate the strain’s potential for practical application. To confirm this potential, further in vitro and in vivo studies are required. Among its applications, it would be interesting to investigated whether the lactobacteria could be formulated as an orodispersible tablet for use in the oral cavity against OC and/or whether the CFS could be used to help maintain dental protheses without *Candida* colonization.

## 4. Materials and Methods

### 4.1. Subjects

In the cohort of students from IUCS-CESPU, 38 students were recruited. All the volunteers agreed to be part of the study by signing the informed consent form that was previously approved from the IUCS Ethics committee (N/Ref.ª. CE/IUCS/CESPU-19/21). The purpose and content of the study were explained fully to all subjects. All participants were examined by the same examiner, and their eligibility to participate in the study was assessed. The inclusion details of the volunteers were the following: caries-free, non-smokers, no history of systemic disease, and no antibiotic therapy for one month prior. Clinical oral examination and X-ray (bitewing) were performed to check for caries between teeth. In addition, anamnesis was also performed.

### 4.2. Saliva Sampling and Isolation of the Lactobacillus

Saliva samples were collected from the eligible subjects using the methodology adapted from Rossoni et al. (2018) [22], using oral rinses of 10 mL of phosphate-buffered saline (PBS, 0.1 M, pH = 7) for 1 min. Then, the samples were centrifuged for 10 min at 5000 rpm (Heraeus Megafuge 16, Thermo Scientific, Waltham, MA, USA), the supernatant rejected, and 2.5 mL of PBS added to the pellet. The samples underwent tenfold serial dilutions, and 100 μL aliquots of each dilution were plated onto Rogosa agar (Milipore, Burlington, MA, USA) using the conventional pour plate method. The plates were then incubated for 72 h at 37 °C with 5% CO_2_. The characteristic discoid colonies on Rogosa agar were selected and subjected to Gram staining and catalase assay. Only Gram-positives and catalase-negatives were isolated for further studies. Stocks were prepared and stored at –80 °C with Man–Rogosa–Sharp (MRS) broth (Milipore, Burlington, MA, USA) medium supplemented with 20% glycerol.

### 4.3. Microorganisms

The ATCC (Manassas, VA, USA) reference strains were purchased, and the clinical oral isolates of *Candida* belonged to our mykotheke (Table 3).

### 4.4. Preliminary Screening of the Oral Isolates

For the selection of the oral isolates with the best performance, a preliminary screening assay was performed, as described in Rossoni et al. (2018) [22], using the reference strains of *C. albicans*, *C. tropicalis*, *C. parapsilosis*, and *C. glabrata*. All *Candida* spp. strains were cultured for 18 h at 37 °C in yeast extract peptone dextrose (YEPD) broth (Millipore, Burlington, MA, USA, USA). The oral isolates were cultured in MRS broth for 24 h at 37 °C with 5% CO_2_. The optical density of the suspensions of *Candida* and lactic acid bacteria was determined using a spectrophotometer and adjusted to a concentration of 10^7^ cells/mL.

The amount of cells in the inoculum was confirmed, plating in Sabouraud dextrose agar (SDA) (Difco, Franklin Lakes, NJ, USA) with chloramphenicol (0.05 g/L) for *Candida* and MRS agar (Difco, NJ, USA) for oral isolates. Then, 250 μL of a *Candida* strain suspension and 250 μL of a bacteria suspension were combined with 1.5 mL of brain heart infusion (BHI) broth (Millipore, Burlington, MA, USA) and incubated at 37 °C for 24 h. In the negative control group, bacteria suspensions were replaced with phosphate-buffered saline (PBS). After incubation, the cultures were diluted, plated on SDA supplemented with chloramphenicol (0.05 g/L), and incubated for 48 h at 37 °C, and *Candida* colony-forming units (CFUs/mL) were determined.

### 4.5. Antifungal Activity Against Planktonic Cells of Candida (MFC)

The activity of planktonic cells of *Candida* was determined solely on the reference strains using an adapted methodology from the EUCAST guidelines [25]. Briefly, *Candida* spp. were cultured in SDA with chloramphenicol (0.05 g/L) agar plates for 24 h at 37 °C (fresh plates). The inoculum was prepared according to Rodrigues et al. (2018) [76], by suspending five distinct colonies, ≥1 mm diameter from 24 h cultures, in at least 3 mL of PBS. The cell density was adjusted to the density of a 0.5 McFarland standard, adding sterile PBS, as required, to yield a yeast suspension of 1–5 × 10^6^ CFUs/mL. The working suspension was prepared by a dilution of the standardized suspension in RPMI-1640 (Sigma-Aldrich, Roswell Park, St. Louis, MO, USA) to yield 1–5 × 10^5^ CFU/mL. The bacteria were inoculated directly from the stock culture in MRS broth and incubated for 24 h at 37 °C with 5% CO_2_. Different concentrations of bacteria suspensions (–10^2^ to 10^8^ CFU/mL) were used by diluting with MRS broth. The bacterial concentration was confirmed by plating on MRS agar and then incubated at 37 °C with 5% CO_2_ for 48 h. A 96-well plate was inoculated with 100 μL of each suspension, i.e., yeast and bacteria, for 24 h at 37 °C. To determine the minimum fungicidal concentration (MFC) of the isolate, CFUs were determined (for each condition). The results were calculated by Log_10_ CFU/mL of *Candida* reduction and presented as CFU/mL of bacteria [7,76].

### 4.6. Preparation of Cell-Free Supernatant

The supernatant was extracted according to Yu et al. (2015) [77] and Rossoni et al. (2018) [22], with minor modifications. Accordingly, *L. reuteri* were incubated in MRS broth for 24 h at 37 °C with 5% CO_2_. After, the inocula were centrifuged for 10 min at 5000 rpm (Heraeus Megafuge 16, Thermos Scientific, Waltham, MA, USA), and the supernatant was filtered using a 0.22 μm pore-size sterile syringe filter (Millipore, USA) and stocked at −20 °C if not used immediately.

### 4.7. Activity Against Candida Biofilms

For the biofilms’ formation, the methodology used was adapted from EUCAST guidelines [25]. All *Candida* strains were grown on SDA with chloramphenicol plates and incubated at 37 °C for 24 h. A pre-inoculum was obtained in 50 mL of SDB and incubated at 37 °C for 16 h. Then, the *Candida* suspension was centrifuged at 3000× *g* for 10 min at 4 °C and washed two times in PBS; the concentration of each *Candida* spp. was adjusted to 1 × 10^5^ cells/mL in RPMI-1640 using a Neubauer chamber. A volume of 200 μL of standardized cell suspensions was plated into selected wells of 96-well polystyrene microtiter flat-bottomed plates (Orange Scientific, Braine-l’Alleud, Belgium) and incubated at 37 °C, with rotation of 120 rpm for 24 h. After that, 100 μL of the *Candida* suspension was removed along with 100 μL of *L. reuteri* AJCR4 suspension (prepared as described in Section 4.5) at different concentrations (10^8^, 10^7^, and 10^6^ CFU/mL). To evaluate the supernatant, 100 μL undiluted CFS and CFS diluted by ratios of 1:10 (CFS 1:10) and 1:100 (CFS 1:100) with MRS broth were added to the *Candida* suspension. Controls were obtained by replacing the bacteria suspension or CFS by 100 μL of MRS broth. The plates were incubated for an additional 24 h at 37 °C with 120 rpm. After, the medium was aspirated, and biofilms were washed once with 200 μL of PBS to remove non-adherent cells. Then, biofilms were scraped from the wells, and the suspensions were vigorously vortexed for 2 min to disaggregate cells from the matrix. Serial decimal dilutions in PBS were plated on SDA supplemented with chloramphenicol and incubated for 24 h at 37 °C in order to determine viable *Candida* cells. The results are presented as Log_10_ CFU/mL. For determination of the minimum biofilm eradication concentration (MBCEC), the results were calculated by Log_10_ CFU/mL of *Candida* reduction and presented as CFU/mL of bacteria [25,76].

### 4.8. Inhibition of the Adherence of Candida spp. to Acrylic Denture Resins

To make the acrylic resin discs, a cast matrix was created with silicone fluid for duplication (UGIFORM 25™, Seyssinet-Pariset, France), with a metal disc that had the following form and dimension (⌀23 × 2 mm). To create he acrylic resin specimens’ discs using a self-curing polymethyl methacrylate acrylic (PMMA), a mixture of polymer (powder) and monomer (liquid) was made according to the manufacturer instructions (Interacryl Cold, Interdetnt^®^, Celje, Slovenia) and used to fill the silicone matrix. After complete polymerization, the discs were removed from the matrix, a visual inspection was performed, and small adjustments were carried out to ensure that the acrylic specimens were standardized. The discs were then dipped in sterile distilled water for 5 days, with daily changes of water to allow release of the residual monomer. For disinfection of the discs, isopropyl alcohol 70% was used. The discs were immersed for 3 min and were removed with sterile tweezers and left to dry completely in a sterile environment. Next, the acrylic specimens were placed in a 6-well plate, and *Candida* biofilms (*C. albicans* SC5314, *C. tropicalis* ATCC750, *C. parapsilosis* ATCC22019, and *C. glabrata* ATCC2001) were induced as described before (Section 4.7). After 24 h, CFS was added to the wells, MRS broth medium was added to the control group, and incubation followed for 24 h at 37 °C, under 120 rpm rotation. To liberate the biofilms from the discs, these were transferred to a 50 mL falcon tube containing 4 mL of PBS and submitted to five cycles of ultrasounds for 30 s followed by vortexing for 30 s. Then, the suspensions were diluted and plated on SDA with chloramphenicol (0.05 g/L) and incubated for 24 h at 37 °C for CFU counts.

### 4.9. Co-Aggregation and Auto-Aggregation Evaluation

For the determination of co-aggregation, an adapted protocol from Jørgensen et al. [53] was used. *L. reuteri* AJCR4 was inoculated in MRS broth and incubated at 37 °C for 24 h with 5% CO_2_. At the same time, *Candida* strains were grown in SDB for 16 h at 37 °C. After, both bacteria and yeast were centrifuged for 10 min and 3000× *g* at 4 °C and then washed three times with PBS and resuspended in fresh PBS; the optical density (OD) was adjusted to 0.5 at 600 nm using a spectrophotometer. Identical volumes (1.0 mL) of the *Limosilactobacillus* and *Candida* strains were mixed and incubated aerobically at 37 °C. At 1, 2, and 4 h, the OD was measured, and co-aggregation was calculated using the following equation:

$$\%\;\text{co-aggregation}=\frac{{OD}_{0}-{OD}_{h}}{{OD}_{0}}\times 100$$

where OD_0_ is the absorbance of the mixed suspension at 0 h, and OD_h_ is the absorbance of the mixed solutions at different time points: 1, 2, and 4 h.

For the auto-aggregation ability of *L. reuteri* AJCR4, a methodology was adapted from Ali et al. [54]. The bacteria were prepared and OD adjusted as described above. The *L. reuteri* AJCR4 cell suspension (2.0 mL) was incubated at 37 °C, and for the different time periods of 1, 2, and 4 h, the OD was measured, and the percentage of auto-aggregation was calculated using the following equation:

$$\%\;\text{auto-aggregation}=\frac{{OD}_{0}-{OD}_{h}}{{OD}_{0}}\times 100$$

where OD_0_ is the absorbance of the *L. reuteri* AJCR4 suspension at 0 h, and OD_h_ is the absorbance of the *L. reuteri* AJCR4 at different time points: 1, 2, and 4 h.

### 4.10. Effect of pH on Antifungal Activity

The pH of the cell-free supernatant of *L. reuteri* AJCR4 was adjusted to 7.0 using 5 mol/L sodium hydroxide (NaOH) [77]. The resultant supernatants were used in the assays for biofilm formation with the four reference strains of *Candida.*

### 4.11. Effect of High-Temperature Treatment on CFS Antifungal Activity

To evaluate if high temperature would affect the antifungal activity, the CFS was heated to 100 °C for 30 min. After cooling, the CFS was used in the assays for biofilm formation [77].

### 4.12. Effect of Catalase and Peroxidase on Antifungal Activity

To evaluate both catalase and proteinase k effects, the pH of the CFS was first adjusted to 7.0 using 5 M NaOH to neutralize acidity. Then, one microliter of each enzyme, namely peroxidase (5220 U/mg) (Sigma-Aldrich, USA) or proteinase k (30 U/mg) (Sigma-Aldrich, USA), was added and incubated for one hour at room temperature. The enzymes were then heated to 65 °C for 30 min to stop the enzymatic reaction [60] and then stocked at −20 °C if not immediately used.

### 4.13. Statistical Analysis

The resulting data of the experiments are presented as the mean value along with the standard deviation. GraphPad Prism (version 9.5.0, Boston, MA, USA) was used to assess statistical significance. For the antifungal activity against planktonic cells and biofilm of *Candida* assays, a two-way analysis of variance (ANOVA) was performed using Dunnett’s multiple comparisons posttest. For the acrylic resin discs and the washed cells assays, the posttest utilized to identify significant differences between the means was Tukey’s multiple comparisons test, with the significance level set at *p* < 0.0001. In the co-aggregation assay, a two-way analysis of variance (ANOVA) using Šidák multiple comparisons posttest was utilized to identify significant differences between the means, with the significance level set at *p* < 0.0001. All experiments were conducted three times in duplicate on separate occasions.

## 5. Conclusions

This work demonstrates that *L. reuteri* AJCR4 cells and supernatant have potential for use in prophylaxis or as treatment of OC, though the in vitro biofilm disruption varied among *Candida* spp. Even though this a preliminary study, the results obtained point to a promising strategy against *Candida* biofilms. Nonetheless, more studies need to be conducted to further assess these effects. Therefore, it is essential that this particular strain be subjected to further exploration with regard to its true probiotic effect and safety before considering a practical application. For instance, *L. reuteri* AJCR4 should be tested against a wider range of oral isolates and other species of *Candida*. Furthermore, the susceptibility of *Candida* biofilms to CFS should be a focus of further investigation, with the aim to explore the application of the latter in cases of denture stomatitis. Also, the long-term effects of this bacteria on the oral *Candida* colonization must be assessed. Exploration of the persistence of the probiotic effects, risk of infection, and its influence on the host immune responses is also necessary.

## Figures and Tables

**Table 1:** MFC values of *L. reuteri* AJCR4 for *Candida* spp.

	*L. reuteri* AJCR4 MFC (CFU/mL)
*C. albicans* SC5314	10^8^–10^7^
*C. tropicalis* ATCC750	10^6^
*C. parapsilosis* ATCC22019	10^8^
*C. glabrata* ATCC2001	10^7^

Abbreviations: MFC, minimum fungicidal concentration; CFU, colony-forming units.

**Table 2:** MBEC values of *L. reuteri* AJCR4 and cell-free-supernatant for *Candida* spp.

*L. reuteri* AJCR4 MBEC (CFU/mL)			
Species	Strain	MBEC (CFU/mL)	MBEC–S
*C. albicans*	SC5314	10^8^	CFS
	H37	10^8^	CFS
	H43	10^8^	CFS
	MYK 2760	10^8^	CFS
*C. tropicalis*	ATCC750	10^8^	CFS
	C7	10^8^	CFS
*C. glabrata*	ATCC2001	10^8^	-
	15	10^8^	CFS
	H49	10^8^	CFS
*C. parapsilosis*	ATCC22019	10^8^	CFS

Abbreviations: MBEC, minimum biofilm eradication concentration; CFU, colony-forming unit.

**Table 3:** Species and origin of *Candida* strains used in the study.

Species	Strain	Country	Origin	Reference
*C. albicans*	SC5314	Reference strain	-	ATCC
	H37	Brazil	Oral	[46]
	H43	Brazil	Oral	[46]
	MYK 2760	Slovakia	Oral	[2,75]
*C. tropicalis*	ATCC750	Reference strain	-	ATCC
	C7	Brazil	Oral	Collection strain
*C. glabrata*	ATCC2001	Reference strain	-	ATCC
	15	Slovakia	Oral	[2]
	H49	Brazil	Oral	[46]
*C. parapsilosis*	ATCC22019	Reference strain	-	ATCC

Abbreviations: ATCC, American Type Culture Collection.

## Data Availability

The data presented in this study are available in this article.
